# Detection of Periapical Lesions Using Artificial Intelligence: A Narrative Review

**DOI:** 10.3390/diagnostics16020301

**Published:** 2026-01-17

**Authors:** Alaa Saud Aloufi

**Affiliations:** Department of Restorative Dental Sciences, Endodontic Division, College of Dentistry, Taibah University, Madinah 42353, Saudi Arabia; asoufi@taibahu.edu.sa; Tel.: +966-541600081

**Keywords:** artificial intelligence, deep learning, convolutional neural networks, periapical diseases, radiography, root canal therapy

## Abstract

Periapical lesions (PALs) are a common sequela of pulpal pathology, and accurate radiographic detection is essential for successful endodontic diagnosis and treatment outcome. With recent advancements in Artificial Intelligence (AI), deep learning systems have shown remarkable potential to enhance the diagnostic accuracy of PALs. This study highlights recent evidence on the use of AI-based systems in detecting PALs across various imaging modalities. These include intraoral periapical radiographs (IOPAs), panoramic radiographs (OPGs), and cone-beam computed tomography (CBCT). A literature search was conducted for peer-reviewed studies published from January 2021 to July 2025 evaluating artificial intelligence for detecting periapical lesions on IOPA, OPGs, or CBCT. PubMed/MEDLINE and Google Scholar were searched using relevant MeSH terms, and reference lists were hand screened. Data were extracted on imaging modality, AI model type, sample size, subgroup characteristics, ground truth, and outcomes, and then qualitatively synthesized by imaging modality and clinically relevant moderators (i.e., lesion size, tooth type and anatomical surroundings, root-filling status and effect on clinician’s performance). Thirty-four studies investigating AI models for detecting periapical lesions on IOPA, OPG, and CBCT images were summarized. Reported diagnostic performance was generally high across radiographic modalities. The study results indicated that AI assistance improved clinicians’ performance and reduced interpretation time. Performance varied by clinical context: it was higher for larger lesions and lower around complex surrounding anatomy, such as posterior maxilla. Heterogeneity in datasets, reference standards, and metrics limited pooling and underscores the need for external validation and standardized reporting. Current evidence supports the use of AI as a valuable diagnostic platform adjunct for detecting periapical lesions. However, well-designed, high-quality randomized clinical trials are required to assess the potential implementation of AI in the routine practice of periapical lesion diagnosis.

## 1. Introduction

Apical periodontitis is an inflammatory condition mainly caused by bacterial infection in the root canal system. Radiographic evaluation is typically employed for its detection, where it appears as periapical radiolucencies, commonly referred to as periapical lesions [1]. Periapical lesions (PALs) represent one of the most common pathologic lesions of the jaws. The presence of PALs on the radiographic imaging is an important prognostic factor in determining the outcome of root canal treatment [2]. In a prospective study investigating factors affecting the outcomes of nonsurgical root canal treatment, the preoperative absence of a periapical lesion was found to improve periapical healing significantly, and in the presence of a periapical lesion, the smaller its size, the better the treatment prognosis [3]. From a clinical viewpoint, effective management of PALs relies on the clinician’s ability to identify them on radiographic imaging, estimate lesion extent, and monitor their healing over time. In everyday practice, clinicians mainly use intraoral periapical radiographs (IOPAs) and panoramic radiographs (OPGs) to assess PALs. Cone-beam computed tomography (CBCT) is used as an additional tool when a three-dimensional evaluation is necessary [4,5].

In practice, IOPA is the standard method for locating periapical lesions. They assist with diagnosis, treatment assessment, and post-treatment follow-up. However, the two-dimensional (2D) nature of periapical radiographs introduces anatomical overlap, geometric distortion, and a limited ability to detect early or small lesions [6,7]. Cone-beam-computed tomography (CBCT) has improved diagnostic accuracy by offering three-dimensional (3D) views of periapical structures. This imaging technique provides much higher radiographic accuracy and detail. Research has shown that, in the assessment of periapical lesions, CBCT can identify 60.9% to 91.3% of lesions, whereas conventional periapical radiographs detect only 39.5% to 69.5% [8,9]. While CBCT offers 3D images, its higher radiation dose, cost, and limited availability make two-dimensional panoramic radiographs (orthopantomograms, OPGs) the preferred first diagnostic tool in most dental settings [10]. OPGs provide a comprehensive view of the jaws and teeth, enabling detection of periapical radiolucencies across multiple teeth in a single image. The diagnostic accuracy of OPGs in PALs detection remains low, with a sensitivity of about 34% [11]. Nonetheless, OPG imaging has high specificity and positive predictive value (PPV) for detecting PALs, and its diagnostic accuracy relies heavily on lesion location [11]. Therefore, OPG results often need to be confirmed with IOPA or CBCT if there remains a clinical suspicion. Observer-dependent factors play a crucial role in the identification of PALs in panoramic, periapical, and CBCT radiographs. These factors include diagnostic experience, fatigue, and bias that may introduce variability in radiograph interpretation. Moreover, describing, interpreting, and recording radiological observations can be a time consuming and resource demanding process that requires substantial professional expertise [12]. To improve diagnostic efficiency and accuracy, dentists today are increasingly using artificial intelligence (AI) technologies. The idea of AI was first introduced in the 1950s. It describes the creation of machines that can perform tasks usually associated with human intelligence [13]. In this field, machine learning (ML) is a discipline that concentrates on algorithms capable of identifying and interpreting statistical patterns within data. Among the different machine learning methods, neural networks (NNs) have shown remarkable effectiveness. They often outperform traditional algorithms when handling complex data types such as images or natural language [14]. Neural networks (NNs) are capable of simulating human cognitive processes such as problem solving, learning, and decision-making, and the most frequently utilized types of NNs are artificial neural networks (ANNs), convolutional neural networks (CNNs), and recurrent neural networks (RNNs) [14].

**Figure 1:**
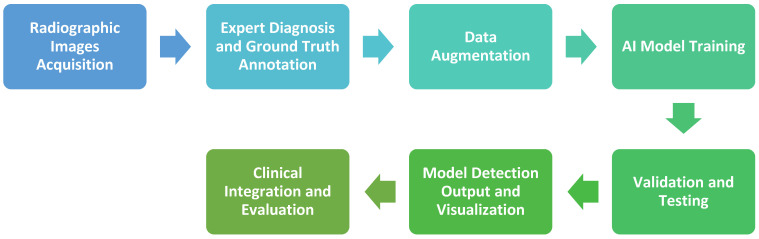
Generalized workflow for studies on AI-based detection of periapical lesions across CBCT, panoramic, and periapical radiographic images.

When looking at published research on assessing artificial intelligence for detecting PALs, a common methodological workflow can be identified and shown in Figure 1. The process begins with image collection (commonly retrospective) and expert annotation, followed by dataset preparation, optional data augmentation, and model training using CNN-based architectures. Performance is validated on unseen data, and detection outputs are reviewed by specialists for clinical reliability.

In their recent systematic review, Pul and Schwendicke included 24 studies published up to 2023 and confirmed the high overall sensitivity and specificity of AI in detecting periapical radiolucencies (PARLs) [15]. The primary studies were highly heterogeneous in imaging modality, reference standards, and outcome metrics, precluding a valid meta-analysis and limiting direct operating-point guidance for clinical use. Likewise, an earlier meta-analysis by Sadr et al. (2023) showed promising accuracy but noted bias and inconsistent reporting across studies [16]. Previous narrative reviews spanning endodontics and dental radiology broaden the scope across modalities but still primarily synthesize outcome metrics and provide little stratification by clinically relevant moderators (e.g., lesion size, tooth type/arch, root-filling status) or a consistent appraisal of reader-study outcomes [17,18,19,20].

Bansal et al.’s scoping review included twelve studies that compared AI performance in detecting PALs across periapical, panoramic, and CBCT radiographs, reporting the highest mean sensitivity on periapical radiographs and the highest specificity/AUC on CBCT, with panoramic radiographs being the lowest [20]. However, it pooled a small number of heterogeneous studies that investigated different types of AI platforms, and it did not synthesize reader-study outcomes, so its guidance for real-world application is limited. Our review complements prior syntheses by grouping studies by imaging modality and organizing the evidence around clinically relevant moderators (i.e., lesion size, tooth type, and anatomical surroundings, root-filled vs. non-root-filled teeth) rather than only reporting pooled metrics, and it integrates reader-study evidence to show when AI assistance changes human performance and the time to a decision. Therefore, this review seeks to bring together the most recent studies that have explored how AI systems perform in identifying periapical lesions on different types of dental images, including periapical, panoramic, and cone-beam computed tomography scans.

## 2. Methods of Literature Selection, Data Collection, and Synthesis

A narrative review was conducted of studies published between 2021 and 2025 that employed AI, machine learning, or deep learning algorithms to detect periapical lesions on periapical, panoramic, and cone-beam computed tomography (CBCT) images. This review focuses on ML/DL computer-vision systems (predominantly CNN-based) for radiographic periapical lesion detection. A comprehensive electronic search was conducted across PubMed/MEDLIN and Google Scholar, supplemented by manual screening of the reference lists of relevant articles. Search terms were combined using MeSH terms and included variations of: “periapical disease”, “artificial intelligence”, “deep learning”, “convolutional neural networks”, “X-rays”, “Cone-Beam Computed Tomography”, and “panoramic radiography”. All titles and abstracts were screened by a single reviewer, who also conducted the full-text eligibility assessment of potentially relevant studies.

Titles and abstracts were screened for relevance, after which full-text assessments were conducted on studies that appeared to meet the eligibility criteria. The inclusion requirements were original research; imaging data derived from humans or human sources in vivo studies; the reporting of at least one diagnostic performance metric (e.g., sensitivity, specificity, accuracy) of AI in the detection of PALs; and papers in English. Exclusion criteria were papers on AI performance in detecting dental findings other than PALs, animal studies, in vitro studies and articles not available in English. Data extracted from the included studies are bibliographic information, such as the author and year, imaging modality (IOPA, OPG, or CBCT), type of AI model, number and expertise of annotators and dataset characteristics (including sample size and reported data subgroups such as tooth type, root-filled status, and lesion size). Additionally, it included diagnostic performance results, such as sensitivity, specificity, and accuracy, along with clinician annotation time and performance aided by an AI model when available. Owing to marked heterogeneity in study design, datasets, outcome measures, and AI architectures, no quantitative meta-analysis was undertaken.

## 3. Results

**Figure 2:**
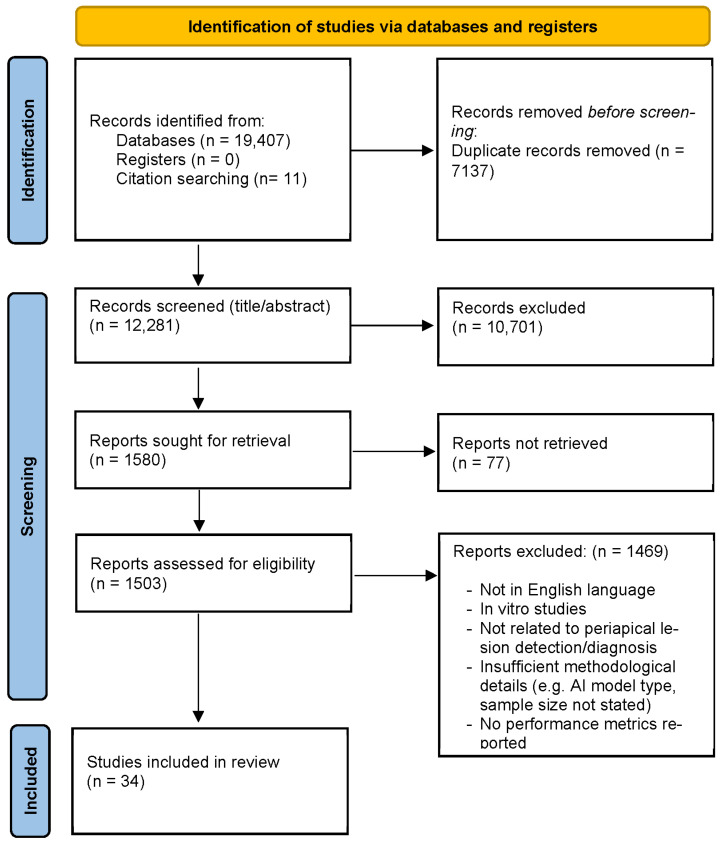
Selection process flowchart.

The electronic database searches yielded 19,407 records (Figure 2), with an additional 11 records identified through citation searching. After removal of duplicates, and irrelevant titles and retrieving full text 1503 records remained and assessed for eligibility. Following full-text review, 1469 articles were excluded with reasons, resulting in 34 studies included in the review synthesis.

The papers were published between January 2021 and July 2025. The selected studies report data from three radiographic modalities: IOPA, OPG, and CBCT. The AI models used were mostly CNN platforms.

### 3.1. AI-Based Systems Performance for Detecting Periapical Lesions Using Panoramic Images (OPG)

Current research demonstrates that AI and deep learning models show considerable promise for detecting periapical lesions on OPGs, though performance varies significantly across studies and clinical contexts.

Thirteen OPG radiograph-based studies were identified. Table 1 summarizes research on AI-based systems for detecting periapical lesions on OPG images and their diagnostic performance metrics.

Panoramic images provide a comprehensive view of both jaws, facilitating the screening of periapical pathology with minimal radiation exposure [21,22]. When diagnosing periapical lesions on panoramic radiographs, even experienced clinicians tend to show high specificity (~95.8%) yet low sensitivity (~34.2%) compared with CBCT [11].

Kim et al. developed an AI-driven system for detecting five common tooth-related diseases, including periapical radiolucency, on 10,000 panoramic images from 50 dental clinics across Korea. The authors noted that Fast R-CNN effectively located periapical radiolucencies and reduced noise and false detections when compared to ResNet and Inception models [23]. Also, the model could process an OPG image in 2 min, thereby significantly reducing diagnostic and treatment-planning time. Ba-Hattab et al. studied a two-step AI system (Faster R-CNN to locate periapical areas, Inception-v3 to classify them as healthy or as periapical lesions) on 713 panoramic radiographs and found it both accurate and fast, analyzing each panoramic image in about two seconds [24]. Similarly, Çelik et al. investigated deep-learning AI systems for PALs identification on panoramic radiographs [25]. They noted that although expert clinicians achieved near-perfect accuracy on the external test set, they required about 24–28 min per batch. In contrast, the deep-learning models generated results in about a second, highlighting a substantial speed advantage of automated methods [25]. Güneç et al. studied how well an artificial intelligence (AI) system performs compared to junior dentists in finding periapical lesions on panoramic radiographs [26]. They found that the AI system (DentisToday) outperformed all three junior dentists and demonstrated higher diagnostic accuracy, reliability, and consistency in identifying periapical pathology [26].

Among studies highlighting the shortcomings of AI interpretation of OPG images, Kazimierczak et al. evaluated the commercial Diagnocat platform on a paired OPG–CBCT cohort. They showed that AI demonstrated significantly higher sensitivity in detecting PALs in CBCT images than in OPG images [27]. The results suggest that the AI tool struggles to accurately identify PALs in OPGs, yet it demonstrates a fairly strong diagnostic capability in CBCT analysis. Moreover, the generalization of deep learning models for detecting PALs on panoramic images proved limited, particularly when tested across datasets from different populations and imaging conditions. Krois et al. trained AI models on a single dataset and, when tested on external data, showed a significant drop in performance. The sensitivity decreased from 48.0% to 22.0%, indicating difficulty detecting lesions in new populations [28].

Overall, AI-assisted panoramic imaging could reliably support clinicians in identifying periapical pathology by contributing to faster diagnosis. However, limitations exist in diagnosis accuracy, dataset diversity, generalization, and annotation standards.

**Table 1:** Summary of Papers on Artificial Intelligence Performance in The Detection of Periapical Lesions Using Panoramic Images.

Author (Year)	AI Model	Primary AI Model Task	Sample Number	Sensitivity	Specificity	Accuracy	Data Subgroups	Annotation of PALs
Krois et al. (2021) [28]	U-Net type, Deep CNN	Segmentation	1300 OPG	48.0%	99.9%	NR	With or without root canal fillings and restorations	Four dental specialists and a master reviewer
Bayrakdar et al. (2022) [29]	U-Net (CranioCatch), Deep CNN	Segmentation	470 OPG	70%	NR	NR	None	Three expert dental radiologists
Kim et al. (2022) [23]	Fast R-CNN, Hybrid CNN	Detection + Classification	10,000 OPG	95.3%	89.55	Accuracy > 90%	5 tooth-related diseases: Coronal, proximal, cervical caries, periapical radiolucency, and residual root	Expert maxillofacial radiologist
Zadrożny et al., (2022) [30]	Diagnocat, CNN	Classification	30 OPGs	39%	98%%	NR	Different dental conditions (missing tooth, caries, filling, periapical lesion, periodontal bone loss, etc.)	Three independent evaluators with 12, 15, and 28 years of experience in dentistry
Song et al. (2022) [31]	U-Net (Deep CNN)	Segmentation	180 Lesions (from 100 OPGs)	82.6%, 80.8%, 74% (IoU intersection over union 0.3, 0.4, 0.5)	NR	NR	None	Three oral and maxillofacial radiologists (with >10 years of experience)
Güneç et al. (2023) [26]	DentisToday, CNN	Detection + Classification	500 OPG	97%	63%	NR	Caries and Periapical lesions	Two specialist dentists with 10 years of experience
Ba-Hattab et al. (2023) [24]	Faster R-CNN detector + Inception-v3 classifier, Two-stage CNN	Detection + Classification	713 OPG	84.6%	72.2%	85.6%	None	Three examiners with >15 years of experience
Çelik et al. (2023) [25]	Faster R-CNN, RetinaNet, YOLOv3, SSD, Libra R-CNN, Dynamic R-CNN, Cascade R-CNN, FoveaBox, SABL, ATSS	Object Detection	357 OPG	0.743–0.918 across models (best: YOLOv3 = 0.918)	0.76 (YOLOv3)	0.673–0.812	None	Oral and maxillofacial radiologist with >7 years of experience
İçöz et al. (2023) [32]	YOLOv3 Darknet model	Detection + Classification	306 OPG	98%	56% (Precision)	NR	Maxilla vs. mandible Widened PL/uncertain AP vs. Clearly identified AP	oral and maxillofacial radiologists
Kazimierczak et al. (2024) [27]	Diagnocat, CNN	Object Detection	49 OPG and CBCT from the same patients	OPG: 33.33% CBCT: 77.78%.	OPG: 98.43% CBCT: 99.83%.	OPG: 97.01% CBCT: 99.35%	None	Orthodontist and radiologist, each with >8 years of experience
Boztuna et al. (2024) [33]	U^2^-Net model, Deep U^2^-Net	Segmentation	400 OPG	85.4%	NR	81% (F1-score)	None	A resident in oral and maxillofacial surgery and a dentomaxillofacial radiologist with >6 years of experience
Szabó et al. (2025) [34]	Diagnocat, CNN	Object Detection	357 OPG	78%	100%	89%	Group 1 (caries) Group 2 (coronal restoration) Group 3 (root-filled)	Two dentomaxillofacial radiologists (>10 years and >30 years of experience)
Pul et al. (2025) [35]	DentalXrai Pro, CNN	Detection + Classification	50 OPG	45.8% (AI-aided) vs. 46.0% (unaided).	98.0% (AI-aided) vs. 95.7% (unaided).	93.3% (AI-aided) vs. 91.6% (unaided)	Performance stratified by experience (junior ≤ 10 years, intermediate 11–15 years, senior > 15 years)	Two CBCT experts

NR: Not reported.

### 3.2. AI-Based Systems Performance for Detecting Periapical Lesions Using Intraoral Periapical Radiographs (IOPA)

Fifteen studies investigating AI-based detection of periapical lesions on periapical radiographs were included, with sample sizes ranging from 60 teeth in small study designs to more than 3000 periapical root areas in large retrospective datasets (Table 2) [36,37,38,39,40,41,42,43,44,45,46,47,48,49,50]. The studies employed a range of AI architectures, including conventional CNN classifiers, transfer-learning models, object-detection frameworks (such as Faster R-CNN, YOLOv3, and Mask R-CNN), and segmentation networks like U-Net. Table 1 provides a summary of studies evaluating artificial intelligence systems for detecting PALs on IOPAs.

Issa et al. tested the Diagnocat platform and achieved an overall accuracy of 96.7% in PALs detection [43]. One of the study’s limitations is its small sample size of 20 periapical radiographs, limiting the generalizability of the findings. The study included both endodontically treated and untreated teeth. When compared to studies that investigated the same AI platform on larger sample sizes with a population of more than 300 root-filled teeth or teeth indicated for primary root canal treatment, using CBCT as a reference standard, Diagnocat had lower accuracy (76.3% (root filled) [50]; 78.5% (root unfilled) [48]).

Li et al. aimed to identify apical lesions and separate them from healthy periapical tissue [36]. They used a CNN platform with specific preprocessing methods, including Gaussian high-pass filtering, and reported an accuracy of 92.5%. This highlights the need for image processing to crisp edges prior to AI model training. Chuo et al. expanded this work by applying transfer learning to multiple CNN backbones (AlexNet, GoogLeNet, ResNet50/101) on 460 periapical images; the best configuration achieved an accuracy of 96.2% [42]. The more recent study by Liu et al. compared ConvNeXt to ResNet34 performance in PALs detection and found that ConvNeXt performed better across all evaluation metrics, demonstrating higher sensitivity [47].

Other investigations evaluated models that localize lesions and, in some cases, assign severity scores. Chen et al. implemented a Faster R-CNN on 2900 periapical radiographs to detect decay, periapical periodontitis, and periodontitis, each graded as mild, moderate, or severe [37]. AI performance was strongly dependent on severity, with severe lesions consistently detected more reliably than mild ones. Moidu et al. trained a YOLOv3 network to assign Periapical Index (PAI) scores (1–5) on 3000 periapical root areas [40]. At the binary level (healthy vs. diseased), the system achieved 86.3% accuracy; intermediate categories (PAI 1–5) were more challenging, with PAI scores 2 and 5 exhibiting the least accurate predictions (30% each).

Fatima et al. proposed a lightweight MobileNet-v2 with additional layers to enhance the performance of the Mask-RCNN on a small periapical dataset to segment five types of endo-perio lesions in 516 periapical images [44]. After image enhancement and data augmentation, the model reached an overall accuracy of 94% and the highest sensitivity for primary periodontal lesions (100%).

Across the included studies, AI models demonstrated strong diagnostic performance in detecting PALs on periapical radiographs. Future work should focus on larger, multi-center datasets. It should also include clear external validation and the integration of AI results with clinical information. This will help ensure the safe and effective use of AI in everyday endodontic practice.

**Table 2:** Summary of Papers on Artificial Intelligence Performance in The Detection of Periapical Lesions Using Periapical Radiographs.

Author (Year)	AI Model	Primary AI Model Task	Sample Number	Sensitivity	Specificity	Accuracy	Data Subgroups	Annotation of PALs
Li et al. (2021) [36]	AlexNet/GoogLeNet/VGG19/ResNet50	Classification	460 images	94.87%	90.00%	92.75%	None	Dental experts
Chen et al. (2021) [37]	Faster R-CNN	Object Detection and Classification	2900 PA	50–60%	NR	NR	Mild (<1 mm); Moderate (1–3 mm); Severe (>3 mm)	Manual bounding box labeling by a clinical expert (>5 years’ experience)
Ngoc et al. (2021) [38]	DentaVN	Object Detection	130 radiographs	89.5%	97.9%	95.6%	Teeth without root canal filling (Group I) and teeth with root canal filling (Group II)	Expert dentists
Hamdan et al. (2022) [39]	Denti.AI, Deep CNN	Object Detection	68 periapical radiographs	93.1% (by case)	73.3% (by case)	NR	None	By CBCT confirmation
Moidu et al. (2022) [40]	YOLO version 3, CNN	Detection/Classification	3000 periapical root areas	92.1%	76%	86.3%	PAI scores 1–5 classified as healthy (1–2) vs. diseased (3–5) groups	Three endodontists
Ari et al. (2022) [41]	U-Net CNN (PyTorch library (version 1.4.0))	Segmentation	1169 periapical radiographs	92%	NR	>90%	Multiple dental features segmented (caries, crowns, fillings, root fillings, PALs)	Research assistant (2 years) and dento-maxillofacial radiologist (12 years)
Chuo et al. (2022) [42]	AlexNet, GoogLeNet, ResNet50, ResNet101	Object Detection	490 periapical radiographs	98.5%	93.9%	Up to 96.21%	None	Dentists with >3 years of clinical experience
Issa et al. (2023) [43]	Diagnocat, CNN	Classification/Detection	60 teeth, 20 periapical radiographs	92.30%	97.87%	96.66%	Healthy vs. unhealthy	Oral and maxillofacial radiology expert (>10 years’ experience) and one trainee
Fatima et al. (2023) [44]	Lightweight Mask R-CNN with MobileNet-v2 Backbone	Segmentation	534 periapical radiographs	NR	NR	94%	Five lesion categories (primary endodontic, primary periodontal, secondary endo-perio, combined, etc.)	Expert radiologists and dentists
Nagareddy et al. (2024) [45]	Diagnocat, CNN	Object Detection	30 anonymized digital periapical radiographs	86.5%	88.1%	89.6%	NR	a senior oral-maxillofacial radiology expert + a trainee using the Periapical Index (PAI)
Wu et al. (2024) [46]	YOLOv8, CNN	Object detection + classification	67 original periapical radiographs	95%	NR	NR	Normal/Apical Lesion/Peri-endo Combined Lesion	Roboflow Annotation tool
Liu et al. (2025) [47]	ConvNeXt and ResNet34, CNN	Object Detection	1305 PRs (1044 train, 261 validation, 800 test)	ConvNeXt: 98.49%; ResNet34: 84.38%	ConvNeXt: 84.11%; ResNet34: 78.13%	ConvNeXt: 91.25%; ResNet34: 81.63%	Novice dentists (A/B/C); diagnostic time reduction	Three oral radiologists (≥15 yr experience)
Allihaibi et al. (2025) [48]	Diagnocat, CNN	Detection + Classification	339 teeth	47.9%	95.4%	78.5%	None	Two calibrated endodontists and CBCT analysis
Ibraheem et al. (2025) [49]	Second Opinion, CNN-based CADe system	Object Detection	300 periapical radiographs	86.6%	98.3%	NR	Evaluated 8 conditions (caries, PALs, crowns, restorations, etc.)	Two oral radiologists
Allihaibi et al. (2025) [50]	Diagnocat, CNN	Detection + Classification	376 teeth	67.3% (tooth level); 54.3% (root level)	82.3% (tooth level); 86.7% (root level)	76.3% (tooth level); 78.5% (root level)	Endodontic treatment outcomes (root-filled teeth)	Two experienced endodontists independently annotated; CBCT was used as the reference standard.

NR: Not reported.

### 3.3. AI-Based Systems Performance for Detecting Periapical Lesions Using Cone-Beam Computed Tomography (CBCT)

Few studies were identified about AI systems’ detection of PALs on CBCT scans (Table 3). The introduction of cone-beam computed tomography (CBCT) has dramatically improved the visualization of periapical bone changes, offering higher spatial resolution and volumetric evaluation of lesions [10]. CBCT has been shown to perform better than conventional IOPA in detecting periapical lesions. It serves as the radiographic reference standard for diagnosing apical periodontitis, according to ex vivo histological studies in human cadavers [7,51,52]. However, diagnosing apical periodontitis in root-filled teeth using CBCT can be difficult, as diagnostic accuracy was lower in root-filled teeth than in non-root-filled teeth [52]. The assessment of CBCT datasets remains largely dependent on the operator’s expertise, often requiring considerable time and being prone to both intra- and inter-observer variability, reflecting the subjective nature of image evaluation [53].

With the rise in artificial intelligence (AI), automated CBCT image analysis has become a promising adjunct for PALs diagnosis [54,55,56,57,58]. Table 3 summarizes research on AI-based systems for detecting periapical lesions on CBCT images and their diagnostic performance metrics. The studies show that AI-assisted CBCT interpretation can be an effective tool for detecting PALs, with acceptable diagnostic accuracy ranging from 68% to 98.9% in endodontic practice. Nonetheless, the small number of published research and the variations in AI models used indicate that it is crucial to achieve standardization and carry out larger-scale validation for practical application.

**Table 3:** Summary of Papers on Artificial Intelligence Performance in The Detection of Periapical Lesions Using CBCT Images.

Author (Year)	AI Model	Primary AI Model Task	Sample Number	Sensitivity	Specificity	Accuracy	Data Subgroups	Annotation of PALs
Hadzic et al. (2023) [56]	CNN is composed of SpatialConfiguration-Net and a modified U-Net	Detection and Segmentation	195 CBCT	86.7%	84.3%	NR	Size: Periapical Index Score 1–5	Two senior oral surgeons > 15 years of experience and one junior dentist
Fu et al. (2024) [55]	PAL-Net (3D CNN)	Detection and Segmentation	279 CBCT	97%	NR	NR	Volume mm3	Two endodontists
Chau et al. (2025) [57]	CBCT-SAM without PPR	Detection and Segmentation	185 CBCT	68.31%	99.88%	98.9%	Upper/lower Incisors/canines /molars	Expert maxillofacial radiologist
	CBCT-SAM			72.36%	99.87%	98.9%		
	Modified U-Net			62.21%	99.86%	97.3%		
	PAL-Net			70.98%	99.87%	98.4%		
Allihaibi et al. (2025) [54]	Diagnocat, CNN	Detection	134 molars (327 roots)	93.9%	65.2%	79.1%	Lesion size S1 up to 3 mm S2 3–5 mm S3 > 5 mm	Two experienced endodontists
Calazans et al. (2022) [58]	DenseNet121	Classification	1000 CBCT slices, sagittal and coronal	64.49%	76.34%	70%	-Without lesions -Lesions 0.5–1.9 mm in size -Lesions > 2 mm in size	A single oral and maxillofacial radiologist with 10 years of experience
	VGG16			64.49%	72.04%	68%		
Kirnbauer et al. (2022) [12]	SpatialConfiguration-Net (localisation) + U-Net (segmentation), CNN	Classification and Segmentation	144 CBCT	97.1%	88.0%	97.3%	NR	Semi-automatic weighted total-variation segmentation; manual review and adjustment by oral-radiology surgeon

NR: Not reported.

### 3.4. Effect of Root Canal Filling Materials on AI Performance in CBCT Images Analysis

The presence of root filling material has a pronounced effect on AI-based diagnosis of PALs, particularly on CBCT images. In a CBCT study, the results highlighted that the AI platform showed “significantly reduced performance” in postoperative scans of root-filled molars [54]. It found that the Positive predictive value (PPV) of the AI model decreased dramatically from 71.8% in non-root-filled teeth to 54.2% in root-filled teeth [54]. In another study, in non–root-filled teeth, the AI-driven platform demonstrated lower sensitivity with comparable specificity to clinicians, whereas in root-filled teeth, it showed higher sensitivity but reduced specificity [50]. Similarly, Ngoc et al. tested a CNN model on periapical radiographs and found that Sensitivity was higher for root-filled teeth (96.2%) compared with untreated teeth (82.7%). However, specificity was lower (87.0% vs. 98.6%), respectively [38]. As the authors discussed, these findings could be attributed to the increased radiopacity of filling materials, which enhances the AI-driven platform’s detection capabilities. At the same time, imaging artifacts can compromise CBCT images, leading to increased false positives. Moreover, Root-filled teeth often have additional variables, such as sealer extrusion, post placement, and previous treatment patterns, that increase diagnostic complexity [54].

In conclusion, while AI shows promise for detecting periapical lesions, the presence of root fillings creates significant challenges, reducing specificity and increasing false-positive rates. Future models should be trained on more balanced datasets with a sufficient number of root-filled teeth. They should also include methods to reduce artifacts or use context-aware preprocessing to improve the assessment.

## 4. Cross Studies Trends in AI Performance

### AI Assistance and Clinician Performance

The use of AI in endodontic diagnostic radiology has improved image interpretation and changed how clinicians work with radiographic data. Across CBCT, panoramic, and periapical imaging modalities, multiple studies have evaluated clinician performance, with and without AI support, in detecting periapical PALs.

In Periapical radiograph-based studies, Hamdan et al. used a deep learning system (Denti.AI) to assist eight dentists in detecting PALs on 68 periapical radiographs [39]. With AI assistance, lesion-detection accuracy increased by 8.6%, compared with unaided readings (*p* = 0.005) [39]. Liu et al. reported similar benefits when novice dentists viewed ConvNeXt- and ResNet34-generated outputs during interpretation: the mean AUC improved from 0.88 to 0.94 with ConvNeXt and from 0.88 to 0.91 with ResNet34, accompanied by a reduction in reading time of approximately 20% [47]. In another study, Ibraheem et al. found that the Second Opinion AI system increased both sensitivity and overall accuracy for detecting periapical lesions and other pathologies for both interns and specialists [49]. A direct-comparison study using CBCT as the reference standard found that AI platforms demonstrated higher sensitivity but lower specificity than experienced endodontists. Diagnocat AI system demonstrated higher sensitivity but lower specificity than clinicians at both tooth (sensitivity: 67.3% vs. 49.3%) and root levels (54.3% vs. 43.8%) [50]. In another study, the authors found that Clinicians demonstrated significantly higher accuracy at the tooth level (86.1% vs. 78.5%, *p* < 0.001) and greater sensitivity (65.3% vs. 47.9%, *p* < 0.001) than Diagnocat, while specificity was comparable (46%) [48]. In addition, a diagnostic-accuracy study compared Diagnocat AI model with two experienced radiologists in detecting periapical lesions, the AI achieved an accuracy of 89.6% [45]. The top-performing radiologist recorded accuracy 98.5%, while the second radiologist performed slightly lower (81.7%). Moreover, The AI model demonstrated the highest mean confidence level (86.5% ± 9.18). The authors conclude that AI performance is comparable to experienced radiologists for detecting periapical periodontitis [45].

CBCT-based investigations report comparable trends. Fu et al. proposed a three-dimensional CNN (PAL-Net) to detect PALs [55]. Their findings showed that PAL-Net improved clinicians’ diagnostic ability when used as an assistive tool. Ten junior dentists (<1 year of experience) and ten senior endodontists (>5 years of experience) interpreted CBCT volumes with and without AI support. With PAL-Net assistance, the average AUC for junior dentists increased from 0.89 to 0.94, and for senior endodontists from 0.91 to 0.93. The diagnostic time per 100 cases was reduced by approximately 69 min for juniors and 32 min for seniors, highlighting the dual benefits of improved accuracy and efficiency [55].

Panoramic radiograph studies showed similar trends. In a randomized controlled trial, Pul et al. evaluated dentalXrai Pro on 50 panoramic radiographs, which 30 dentists read [35]. AI assistance increased overall diagnostic accuracy from 91.6% to 93.3% and reduced false-positive identifications, with the most significant gains observed among junior clinicians. Regarding reading time for AI systems of OPGs PALs, a study of 30 OPGs showed an average reading time of up to 2.0 min for human evaluators to prepare a single report, and 8.5 min for human evaluators [30]. Other studies on OPGs reported shorter analysis times of about 1.2 s per radiograph [33] and 2.3 s per radiograph [24]. Moreover, dentists reported slightly higher confidence when assisted than when not assisted, though this finding was not statistically significant [35].

Taken together, these studies show that AI assistance enhances clinician performance in detecting periapical changes by improving diagnostic performance and reducing interpretation time, especially for less experienced practitioners. At the same time, they emphasize that AI should function as a decision-support tool under clinician supervision.

## 5. Influence of Periapical Lesion Size on AI Performance

Lesion size is another key determinant of AI performance, particularly in CBCT studies. Due to differences in lesion size stratification thresholds and measurement units across studies (e.g., linear diameter in mm, area-based measures, categorical grading), generalizing lesion size thresholds into a single unified metric was not feasible. Each study’s original definition of ‘small’ lesions and corresponding stratified metrics are presented in Table 2 and Table 3. Fu et al. validated a 3D CNN (PAL-Net) on CBCT images. They reported an accuracy of over 98% for lesions larger than 2.45 mm^3^ and 87% for lesions smaller than 1.2 mm^3^ [55]. This shows that high performance is possible even for small lesion volumes when networks are carefully improved. However, most studies show reduced sensitivity for early, small lesions. Diagnocat platform showed significantly higher sensitivity for medium (3–5 mm) and large (>5 mm) lesions (96.8% and 92.9%, respectively) compared with small lesions (<3 mm), for which sensitivity dropped to 64.9% (*p* < 0.001) [54]. Calazans et al. similarly stratified CBCT-detected lesions into small (0.5–1.9 mm) and large (≥2.0 mm) categories and found that VGG-16 and DenseNet-121 achieved their best performance on large lesions (accuracy 81.3% and 79.2%, respectively) compared with 66.7% and 66.0% for small lesions, respectively [58].

In a validation study of 195 CBCT scans, Hadzic et al. reported that a CNN-based system missed approximately half of the smallest lesions (PAI score 1, 0.5–1 mm), whereas the sensitivity for lesions greater than 1 mm (PAI scores 2–5) reached 90.4% [56]. Moreover, Chen et al. found that deep networks detected moderate and severe radiolucencies (Moderate (1–3 mm); Severe (>3 mm)) far more reliably than mild or borderline lesions (<1 mm), with higher recall and average precision for advanced disease [37]. These findings match clinical experience. Small changes in the periapical region that are limited to cancellous bone lead to slight differences in radiodensity and only minor involvement of the cortex. This makes detection difficult for both human observers and AI algorithms.

## 6. Influence of Tooth Type and Anatomical Surroundings on AI Performance

Tooth type and nearby anatomy also affect model performance. In both 2D and 3D images formats, AI systems usually perform better with teeth that have simpler anatomy and clear surrounding structures. In a CBCT study, clinicians demonstrated significantly higher sensitivity in detecting PARLa in the first upper molars (U6) than AI models (65.7% vs. 40.0%, respectively). In the posterior maxilla and in the roots near the maxillary sinus, Diagnocat also struggled to differentiate these subtle radiographic features, resulting in lower sensitivity [48]. Likewise, Allihaibi et al. found that the performance of both AI-driven platforms and clinicians in detecting lesions in maxillary molars and their roots was more challenging than in mandibular molars [50]. They linked this difference to interference from the maxillary sinus and anatomical superimposition. This is consistent with Hadzic et al.’s results, which showed that lower jaw periapical lesions had a significantly higher specificity than those in the maxilla. They assume that this decrease in false positive findings for the mandible is due to its better radiological assessability compared to the maxilla [56]. In another analysis, the sensitivity of the AI model in maxillary molars decreased as the root apex approached the maxillary sinus floor: from 100.0% for distant roots (>2.5 mm) to 70.6% for roots <1 mm from the sinus (*p* < 0.01) [54]. Also, İçöz et al. found that the number of roots with AP detected correctly in the mandible was higher than in the maxilla: 169 vs. 56 out of 200 roots, respectively [32]. On the other hand, one study on periapical radiographs showed no statistical differences in sensitivity and specificity among maxillary anterior, maxillary posterior, mandibular anterior, and mandibular posterior teeth [38]. Overlapping anatomical structures, sinus pneumatization, and variable trabecular patterns can mimic or mask periapical radiolucencies, challenging both human readers and AI systems [38]. This relationship between anatomical complexity and AI accuracy suggests that training focused on specific areas or clear tooth-type labels may help improve model performance.

## 7. Conclusions

In conclusion, the evidence suggests that AI-assisted systems are effective in detecting large, distinct periapical lesions. However, their performance drops in more complex cases, such as with root-filled teeth and in small PALs. Moreover, AI systems demonstrated comparable or superior sensitivity to human examiners. Although AI use in endodontics has been encouraging so far, several challenges still prevent its smooth adoption in everyday dental practice. Many researchers have used datasets that are either small or limited, making it hard to apply their results to larger populations. Also, the diagnostic accuracy of AI systems can vary greatly depending on the particular algorithm used. Therefore, it is important to confirm their generalizability through validation studies using larger, representative datasets in randomized clinical trials before fully integrating these tools into endodontic practice. Over-reliance on AI systems, especially when the details are unclear, risks unnecessary treatment.

## Data Availability

No new data were created or analyzed in this study.
